# Synthesis of Poly-Lactic Acid by Ring Open Polymerization from Beer Spent Grain for Drug Delivery

**DOI:** 10.3390/polym16040483

**Published:** 2024-02-08

**Authors:** Snehal R. Vakati, Gary Vanderlaan, Matthew D. Gacura, Xiaoxu Ji, Longyan Chen, Davide Piovesan

**Affiliations:** 1 Department of Bioengineering and Biomedical Engineering, Gannon University, Erie, PA 16541, USA; vakati002@gannon.edu (S.R.V.); ji001@gannon.edu (X.J.); chen084@gannon.edu (L.C.); 2 Department of Biology, Gannon University, Erie, PA 16541, USAgacura001@gannon.edu (M.D.G.)

**Keywords:** poly-lactic acid, sustained release rates, brewer’s spent grain, bacterial fermentation, lactide ring polymerization, metal catalyst, drug-eluting synthetic polymer, biomaterials, trimethoprim

## Abstract

Poly-lactic acid (PLA) is a synthetic polymer that has gained popularity as a scaffold due to well-established manufacturing processes, predictable biomaterial properties, and sustained therapeutic release rates. However, its drawbacks include weak mechanical parameters and reduced medicinal delivery efficacy after PLA degradation. The development of synthetic polymers that can release antibiotics and other medicines remains a top research priority. This study proposes a novel approach to produce PLA by converting Brewer’s spent grain (BSG) into lactic acid by bacterial fermentation followed by lactide ring polymerization with a metal catalyst. The elution properties of the PLA polymer are evaluated using modified Kirby–Bauer assays involving the antimicrobial chemotherapeutical, trimethoprim (TMP). Molded PLA polymer disks are impregnated with a known killing concentration of TMP, and the PLA is evaluated as a drug vehicle against TMP-sensitive *Escherichia coli*. This approach provides a practical means of assessing the polymer’s ability to release antimicrobials, which could be beneficial in exploring new drug-eluting synthetic polymer strategies. Overall, this study highlights the potential of using BSG waste materials to produce valuable biomaterials of medical value with the promise of expanded versatility of synthetic PLA polymers in the field of drug-impregnated tissue grafts.

## 1. Introduction

**Figure 1:**
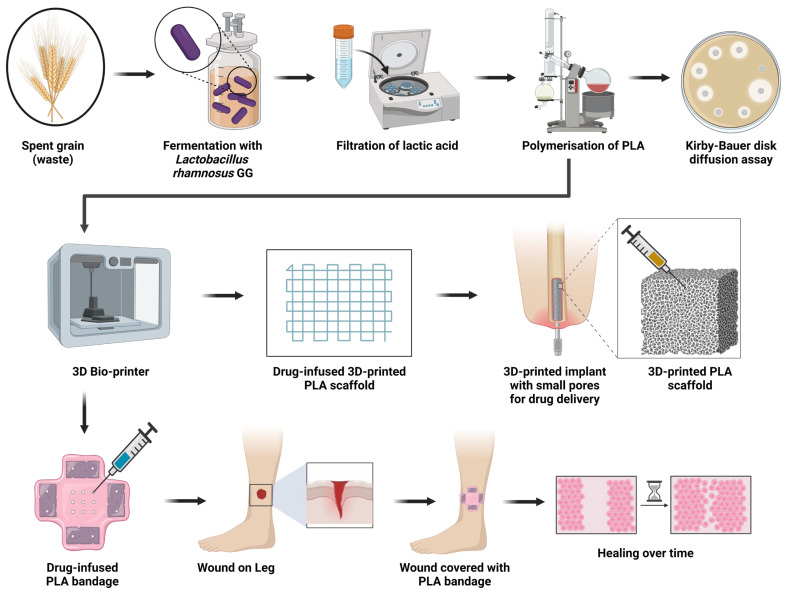
Synthesis of poly-lactic acid (PLA) for biomedical applications. Created with Biorender.com (accessed on 29 January 2024).

Orthopedic surgery-related deep bone infections remain difficult to treat, often acting as sites of localized re-infection. Most orthopedic complications are bacterial infections caused by *Staphylococcus aureus* [1]. Although the penetration of antimicrobials into bone has been thoroughly established, questions remain regarding the kinetics of drug elution rates and the amounts necessary for bone sterilization in vivo. Additionally, it can be challenging to resolve the infectious agent without compromising either structural stability or joint function, as metal or plastic implants can provide pockets conducive to microbial colonization [2,3]. After joint replacement surgery, osteomyelitis is treated by removing the implant, performing surgical debridement, and injecting a drug carrier substance for local antibiotic delivery into the afflicted bone. The most frequently utilized drug delivery matrix is poly-methyl methacrylate (PMMA), which is formed into the required shape and size after being combined with the desired antimicrobial [4]. Unfortunately, PMMA strategies as a drug delivery mechanism must overcome key issues, including poor drug elution qualities, cement dislodging, bone degradation, and the implant’s vulnerability to nidus formation, permitting bacterial colonization. The quantity of PMMA bone cement that must be used to ensure that the implant is firmly secured in place may need to be increased when there is a more severe fracture of the bone. However, doing so might increase the possibility of aseptic loosening and the danger of general thermal necrotic damage [5]. Additionally, PMMA’s polymerization reaction is quite exothermic, which precludes the usage of numerous heat-labile antimicrobials [6]. Taken together, there is a substantive need for novel and effective drug delivery materials and biomedical implants [3,4]. Due to recent developments in 3D-printing technology, alternative biocompatible materials including PLA are an attractive option in orthopedic applications (Figure 1).

PLA is a flexible and biodegradable aliphatic polyester manufactured from renewable resources. The PLA polymer is made of repeating lactate monomers, an organic acid that is naturally formed during lactic acid fermentation involving the reduction of pyruvate by NADH coenzymes [7]. High-purity lactic acid has traditionally been biologically produced by microbial fermentation involving *Bacillus amyloliquefaciens*, numerous *Lactobacillus* spp., *Lactocaseibacillus casei*, *Lactococcus lactis*, and *Streptococcus salivarius* [8]. Each microbial taxon exhibits its own optimal growth parameters and nutritional requirements, with varying levels of lactic acid production [9]. In 2020, beer production was estimated at 1.82 × 10^11^ L worldwide, with a resulting 36.4 million tons of BSG as waste products [10]. BSG is thus an attractive target for numerous upcycling initiatives, including PLA production.

Brewer’s spent grain, which accounts for around 85% of all produced byproducts in the brewing business, is an insoluble residue obtained after wort preparation [11,12]. BSG is a lignocellulosic material composed of the husk pericarp and seed coating that predominantly consists of lignin (28%), arabinoxylans (28%), and around 17% to 28% non-cellulosic polysaccharides [13]. Although BSG is used primarily for livestock feeding, there is great growth potential in using BSG as a nutritional source for lactic acid fermentation. In fact, lactic acid bio-based products offer some of the greatest economic growth potential, as stated in recent reviews [10]. Lactate polymerization of BSG-fed microbes thus offers a potential low-cost biomaterials production strategy.

Biomedical systems are often divided into two categories: permanent and transient. A permanent device, like an artificial knee, should not deteriorate and needs only replacement every 10 years. Sutures, on the other hand, are a temporary solution that ought to dissolve as soon as the tissue that was sewn has recovered from its wound and become strong enough to stand on its own. In the past, the insertion of permanent screws by orthopedic surgeons during repair procedures prohibited all further bone development [14]. New materials, like PLA, have transitory qualities, which means that while they are initially strong and supportive, grafted PLA matrices eventually break down to free up space in the host body for newly formed bone to develop and occupy. The hydrolysis of the ester bond backbone is the principal mechanism by which PLA degrades within the body. Abiotic parameters of PLA, including the pH, temperature, and matrix content of the polymer, all contribute to overall polymeric degradation by host tissues [14]. Additionally, host inflammatory responses can accelerate PLA polymer deterioration rates. This makes it possible to customize the implant for its intended biological use after accounting for these factors.

PLA polymers are an ideal option for transient biomedical applications since the ester bonds in the polymer backbone are amenable to hydrolysis without the need for additional procedures or enzymes and because the breakdown products are non-toxic and minimize the host immune response [15]. A promising avenue for the growth of biomedical engineering is the production of nanomaterials. To improve medication circulation in the body while minimizing harmful side effects, biopolymers can be utilized to encapsulate a wide array of pharmaceuticals [16]. Poly-lactic acid thus plays a crucial role in contemporary industrial technologies, and is employed in bone screws, medical sutures, and dissolvable bandages.

Due to their capacity to be loaded with various medications, biopolymers like poly-lactic acid are excellent platforms as drug delivery systems (DDSs). These DDSs may be transdermal or implantable. The interactions between the PLA polymer and the loaded drug and the diameter of the polymer serve as major determinants of the drug-release requirements for these systems [16]. The PLA polymer, exhibiting macroscopic lengths and small diameters, readily lends itself to both nanoscale DDS types driving the biopharmaceutics and pharmacokinetics specifications of drug molecules for desired clinical effects. This technique also makes it incredibly simple to manufacture, package, and deliver medications via their typical matrix solid forms. Many antimicrobials have the capability to be utilized in drug delivery systems. For example, trimethoprim (TMP) is used in the treatment of various infections and has been delivered using hydrogel prototypes of TMP-impregnated bandages for wound treatment [17]. The present paper presents a research method to test the drug elution properties of an engineered PLA biopolymer.

## 2. Materials and Methods

### 2.1. Brewer’s Spent Grain (BSG) Collection

Depending on the brewing protocol and the style of beer produced, BSG is generally composed of processed grain and water in mass ratios ranging from about 30:70 to 15:85. Empirical exploration of BSG data obtained from several different breweries reveals that the solid BSG fraction, which comprises 15% to 30% of total BSG mass (*w*/*v*), is especially rich in proteins, lipids, cellulose, lignin and a few other components whose concentrations vary depending on the source of material [18]. In this present paper, the BSG was acutely collected as a raw material from local breweries such as Lavery Brewing Company [Erie, PA, USA], a craft microbrewery that specializes in beer but not wine production.

### 2.2. Pre-Treatment of BSGs to Concentrate Nutrient Value

Collected BSG was desiccated for 24 h in a conventional gravity convection oven [Thermo Lindberg Blue M GO1305A, Waltham, MA, USA] at 60 °C to remove excess moisture to minimize deleterious effects on downstream fermentation procedures. Dried bulk BSG is a complex mixture of carbohydrates, proteins, and other nutrients that are excellent caloric inputs for fermentation pathways. Dried BSG was reconstituted by dissolution in sterile water at a solvent-to-solute ratio of 4:1 inside a chemical safety cabinet [Kewaunee Scientific Corporation, Supreme Air Hood, Statesville, NC, USA].

BSG sugar composition varies depending on numerous factors, including the original plant feed, the specific yeast species and strain used to brew alcohol, and also on the specific brewing methodologies, which are often proprietary tradecraft. Fermentable sugars such as maltose, glucose, maltotriose, and fructose are readily found at low levels in BSG [19]. On the other hand, the fibrous component of BSG ranges from ~30–50% and represents a considerable amount of potential nutrients for organisms that can process cellulose and lignin [19]. Cellulose and hemicellulose are polysaccharides that comprise the plant cell wall and dominant dry BSG molecular profiles. Cellulose itself is composed of repeating ß-glucose units and as a polymer is exceptionally difficult for most organisms to hydrolyze as a nutrient source. Hemicellulose is a polymeric polysaccharide comprised of varying mixtures of sugar subunits, including D-xylose, mannose, L-arabinose, galactose, and glucuronic acid [20]. All the above are potential nutritional carbohydrates for lactic acid fermentation by bacteria, except for cellulose, as most lactic acid fermenting bacteria lack operons encoding prokaryotic cellulase enzymes. Additionally, the chemical nutrient composition of BSG varies depending on the brewing process, which is genotypically driven by the precise cultivar strains used to obtain barley source nutrients, the malting and lautering processes during beer formulation, and the overall quality and formulation of the brewing cereal nutrient package, including proper storage and sterile techniques against microbial contamination [21,22,23]. Also, the proprietary recipe behind a particular local brewer’s means of making beer can have an impact on the BSG chemical composition. However, despite these numerous factors, the chemical composition of BSG is typically replete with dietary fiber (i.e., polysaccharides, and, to a lesser extent, oligosaccharides, and monosaccharides), polypeptides, oligopeptides, amino acids, vitamins, minerals, lipids, and polyphenols [21,24]. Our local microbrewery source of BSG did not wish to reveal the specific chemical composition of the various nutrients found within the BSG as an extra precaution to guard against reverse engineering of their beer-making recipes, and we respected the company’s wishes. We thus performed a comparative literature review to gain statistical insights into the precise chemical composition of BSG; doing so revealed a statistically common nutrient profile [25,26,27,28,29,30,31]. Across all examined studies, BSG nutritional content exhibited an average protein amount of 221.5 g ± 27.1 g, average lignin amount of 195.4 g ± 45.9 g, average cellulose amount of 222.2 g ± 24.8 g, mean xylose of 180.3 g ± 20.2 g, and mean arabinan of 77 g ± 9.8 g for every 1 kg of dry BSG weight [25,26,27,28,29,30,31]. BSG mineral content averages 10.7 g Si, 5.2 g P, 3.5 g Ca, 1.9 g S, and 1.9 g of Mg per 1 kg dry BSG [26]. Milligram and trace amounts of minerals are also detectable in dry BSG at average levels of 309 mg Na, 258 mg K, 193 mg Fe, 178 mg Zn, 51 mg Mn, 36 mg Al, 18 mg Cu, 13 mg Ba, 12 mg Sr, and 5.9 mg Cr per 1 kg of dry BSG [26].

**Figure 2:**
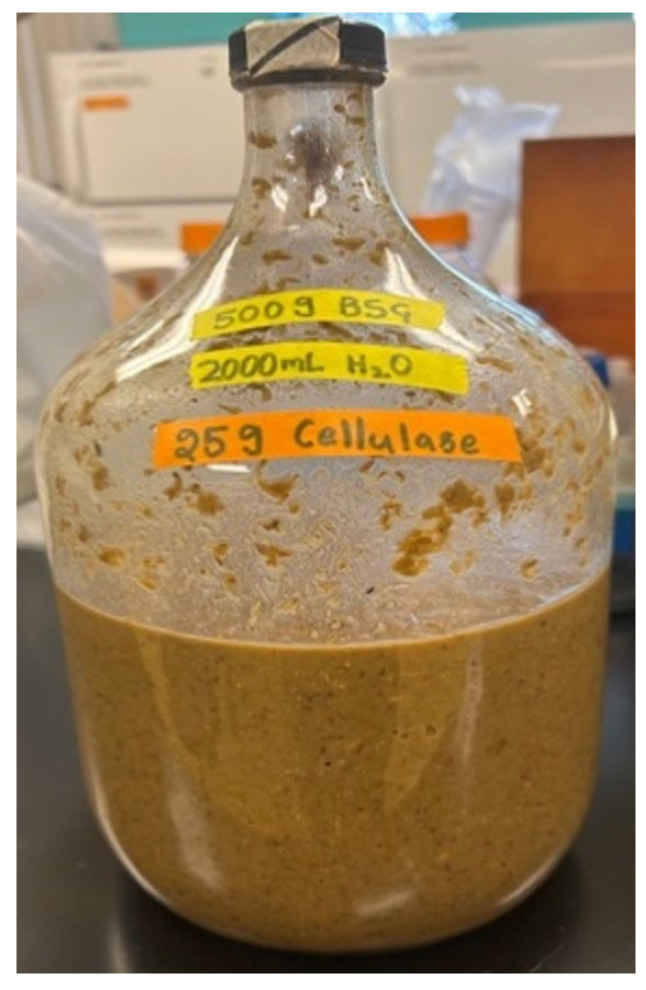
Pre-treatment of Brewer’s Spent Grain (BSG).

Lactic acid yields are reliant on a cadre of variables, including nutritional substrate media, pre-treatment processes such as enzymatic digestion, and post-reactor methodologies, including filtration [32]. Pre-processing of nutrient media typically increases lactic acid production yields by close to an order of magnitude [33]. For example, lactic acid production without enzymatic pre-digestion yielded 5.4 g/L while pre-treatment of nutrient media by enzymatic digestion greatly improved lactic acid yields to 53.1 g/L [33]. Reconstituted BSG was enzymatically treated with cellulase enzyme [Ward’s Science, cat. no. 470300-676, Rochester, NY, USA] digestion for 24 h at 37 °C [VWR Incubator, cat. no. 89511-418, Radnor, PA, USA] to release additional sugar moieties useable by bacterial fermentation pathways (Figure 2). These dissolved, fermentable carbohydrates are collected via filtration by passing enzymatically digested, reconstituted BSG through a standard, sterile cheesecloth in a chemical fume hood [Kewaunee Scientific Corporation, Supreme Air Hood, Statesville, NC, USA]. Solid materials that fail to pass through the cheesecloth are discarded, and the aqueous filtrate is then saved as nutritional media consisting of a simple sugar profile for use in subsequent bacterial lactic acid fermentation.

### 2.3. Bacteriology: Media, Growth, and Staining

All bacteriology work was performed using a sterile biological safety cabinet [NuAire Labgard Class II Type A2 Biological Safety Cabinet, cat. no. NU-540, Plymouth, MN, USA] to maximize axenic conditions and minimize contamination. Luria–Bertani (LB) is a nutrient-rich medium [VWR, cat. no. J106-500G, Radnor, PA, USA] generally used for the growth of non-fastidious bacteria. LB broth and agar was used for *Lactobacillus rhamnosus GG* cultivation. First described in 1967 [34], *L. rhamnosus* strains are short Gram-positive homofermentative non-endospore forming rods that exhibit chain (i.e., strepto) arrangements [35]. Additionally, all Genus *Lactobacillus* contains either facultative anaerobes, aerotolerant anaerobes, or microaerophiles [35]. *Lactobacillus rhamnosus GG* is specifically a gut-isolate strain exhibiting a facultative anaerobic lifestyle exploiting homofermentative pathways and is capable of executing solely lactic acid fermentation in the absence of all other fermentation routes. The LB broth is completely dissolved and left to cool until it reaches an adequate temperature prior to microbial inoculation. The probiotic pill [Supersmart, Los Angeles, CA, USA] containing *Lactobacillus rhamnosus GG* was initially inoculated into a starter culture broth, vortexed to distribute microbial inoculum, and incubated with anaerobic sachets [VWR, cat. no. 90003-642, Radnor, PA, USA] in a Brewer chamber [VWR, cat. no. 90003-636, Radnor, PA, USA] within a standard, microbial growth incubator [VWR, cat. no. 89511-418, Radnor, PA, USA] for 8–12 h at 37 °C without agitation. Following overnight incubation, the *L. rhamnosus GG* starter culture approaches the population log phase of bacterial growth, a stage which greatly helps to improve the fermentation process when used as inoculum due to the actively dividing and thus, metabolically active microbes. The spread-plate methodology was used to isolate pure cultures of *L. rhamnosus GG* from the starter culture broth at serial dilutions of 1:10, 1:100, 1:1000 and 1:10,000. Typically, the 1:1000 diluent yielded optimal pure cultures of *L. rhamnosus GG* in our bacteriological methods.

**Figure 3:**
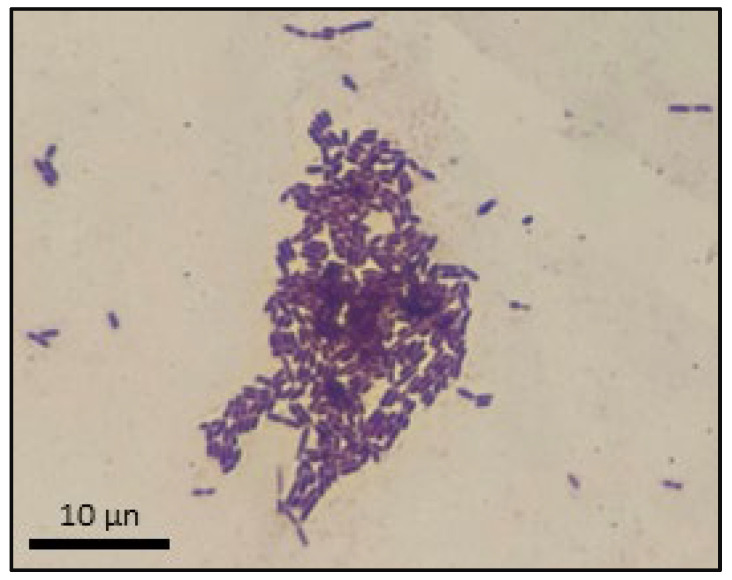
Microscopic analysis of *Lactobacillus rhamnosus GG*.

Pure cultures of *L. rhamnosus GG* were viewed under standard, binocular compound light microscopy [VWR, cat. no. 89404-470, Radnor, PA, USA] using the Gram staining procedure employing typical crystal violet and safranin dyes, iodine mordant, and acetone-alcohol decolorizer treatment times (Figure 3). As an added precaution, we employed an additional streak plate method to confirm pure culture isolation of *L. rhamnosus GG* from probiotic pill starters. Both methods confirmed that sampled probiotic pills contained pure cultures of *L. rhamnosus GG*.

### 2.4. Bacterial Fermentation Procedure

The filtered supernatant (i.e., the filtrate) from the cellulase enzymatic digestion of reconstituted BSG contains a wide berth of dissolved, fermentable sugars [19]. This nutritional filtrate was carefully introduced into a 1-gallon fermenter apparatus [North Mountain Supply, cat. no. 52150000, Mildred, PA, USA] in the presence of pure *L. rhamnosus GG* conveyed via a probiotic pill [Supersmart, Los Angeles, CA, USA] under sterile conditions [NuAire Labgard Class II Type A2 Biological Safety Cabinet, cat. no. NU-540, Plymouth, MN, USA]. This particular microbial species robustly converts simple sugars into lactic acid via fermentation pathways that are optimal in anaerobic growth conditions for 40–76 h at 37 °C [VWR Incubator, cat. no. 89511-418, Radnor, PA, USA] and an initial starting pH of 6.6. Periodic pH measurements were performed to track the efficacy of lactic acid production by measuring subtle decrements in growth media pH until ~4.0. Typically, no further change in pH below 4.0 was seen after 48 h of *L. rhamnosus GG* cultivation in our fermenters, which indicated the completion of lactic acid fermentation. Homofermentative *L. rhamnosus GG* readily converts glucose to pyruvates and then into L-lactate molecules.

### 2.5. Lactic Acid Harvest 

After 48 h of fermentation in a chemical fume hood [Kewaunee Scientific Corporation, Supreme Air Hood, Statesville, NC, USA], our fermenter vessels contained two layers, an upper aqueous layer and a solid bottom layer. Both layers are replete with a complex mixture of L-lactic acid alongside numerous components, including dying microbes and other unwanted biomaterials. The top layer in the fermentation vessel is especially rich in lactic acid fermentation products; this top layer was harvested and a crude, aqueous filtrate was collected using gravity filtration across standard qualitative folded filters [Whatman, cat. no. 10314843, Marlborough, MA, USA] to separate free-floating solids from dissolved solutes (e.g., lactate).

**Figure 4:**
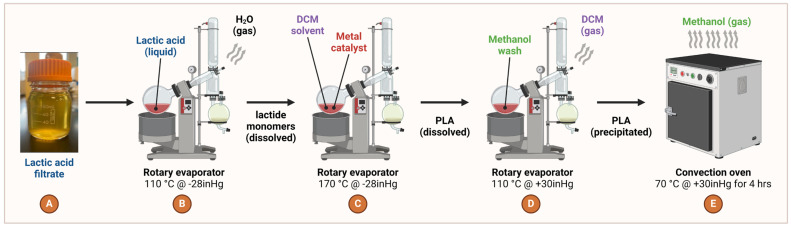
PLA production pipeline. (**A**,**B**) Filtered lactic acid is loaded into a rotary evaporator at high temperature and low pressure to encourage formation of aqueous lactide monomers. (**C**) Dichloromethane (DCM) and a zinc metal catalyst are then injected into the vessel at high temperature and low pressure for aqueous PLA formation. (**D**) A methanol stream is trickled across the PLA at high temperature and standard pressure to precipitate PLA while simultaneously removing DCM. (**E**) Methanol is removed from precipitated PLA under warm heat and standard pressure for many hours. Created with Biorender.com (accessed on 29 January 2024).

The crude filtrate of enriched lactic acid was then ultracentrifuged [Sorvall RC 5B Plus, Waltham, MA, USA] at 10,000 rpm for 15 min using a DuPont SS-34 rotor housing 50 mL ultracentrifuge tubes to further separate solid particles from the aqueous solution. After centrifugation, the supernatant was collected, and the pellet was discarded. To further refine the lactic acid solution, the supernatant was then subjected to vacuum filtration using a 0.45 μm filter [Labfil, cat. no. C0002029, Zhejiang, China] under atmospheric pressure in a 1 L Büchner filtering flask [Fristaden Lab, cat. no. FLP, Chicago, IL, USA] performed in a chemical fume hood [Kewaunee Scientific Corporation, Supreme Air Hood, Statesville, NC, USA]. This vacuum filtration step effectively removes most of the complex cell mass that obstinately remains dissolved in the solution. Following vacuum filtration, the aqueous vacuum filtrate was once again subjected to Sorvall ultracentrifugation at 10,000 rpm for 15 min. The supernatant was recovered following this second ultracentrifugation step to maximize high-purity lactic acid isolation (Figure 4A).

### 2.6. Conversion of Lactate to Lactide

The moisture content in the enriched lactic acid filtrate greatly limits the ability to produce high molecular weight polymers. A 5 L rotary evaporator [TECHTONGDA, cat. no. RE501, Shandong, China] was used to desiccate harvested lactate samples by heating at 110 °C for 1 h in a chemical safety cabinet [Kewaunee Scientific Corporation, Supreme Air Hood, Statesville, NC, USA] at a pressure of −28 inHg (Figure 4B). This treatment can produce lactide (3,6-dimethyl-1,4-dioxane-2,5-dione), as described in [36], which used similar conditions for temperature and pressure (Figure 4B). Lactide acts as an intermediate cyclic ester for PLA production [37]. The present process can produce PLA oligopolymers as well. Lactides are essentially ring forms of lactic acid and act as building blocks that are amenable to efficient polymerization [38]. Also known as lactone, lactide is a cyclic ester derived from lactic acid for subsequent use in dehydration synthesis reactions [38,39]. The lactide moiety comprises two lactic acid monomers that have formed a ring structure, losing water in the process [40]. Lactide formation can be rapidly formed through a condensation reaction where two molecules of lactic acid are dehydrated, and lactide is an important intermediate in the production of PLA [38,39,40,41]. In the presence of a suitable catalyst, lactide undergoes ring-opening polymerization to form PLA [38,39,40,41]. PLA oligomers are short-chain polymers consisting of several lactic acid units linked together (Figure 4C). Unlike lactide, they do not form a cyclic structure but rather a linear or slightly branched chain [38,39,40,41]. They are formed by the polymerization of lactic acid, but the chain length is much shorter compared to high-molecular-weight PLA [38,39,40,41]. The degree of polymerization in oligomers is limited, resulting in a lower molecular weight.

PLA oligomers can have different properties and applications depending on their molecular weight and structure. They might be used as intermediates or precursors in the production of higher-molecular-weight PLA or for specialized applications where shorter chain lengths are beneficial.

In summary, lactide is a cyclic dimer of lactic acid and a precursor for PLA production, whereas PLA oligomers are short-chain polymers made up of lactic acid units. The process of converting lactide to high-molecular-weight PLA involves polymerization, which is a key difference from the formation of PLA oligomers directly from lactic acid.

### 2.7. PLA Production and Characterization by Nuclear Magnetic Resonance (NMR)

A metal catalyst and organic solvent is typically used to polymerize lactide moieties into PLA molecules (Figure 4C). Tin is typically used for PLA synthesis, but zinc can substitute as a metal catalyst (Figure 4C). Polymerization is the process of combining monomers (small molecules) to form a polymer (large molecule). In the case of poly-lactic acid formation, this polymerization process is done in the presence of a zinc metal catalyst [VWR, cat. no. 100209-900, Radnor, PA, USA] and 60 mL of 100% dichloromethane [Macron Fine Chemicals, cat. no. 1593, Center Valley, PA, USA] as an organic solvent at 170 °C under vacuum conditions within a rotary evaporator [TECHTONGDA, cat. no. RE501] located in a chemical safety cabinet [Kewaunee Scientific Corporation, Supreme Air Hood, Statesville, NC, USA] (Figure 4C). PLA is produced by a process called ring-opening polymerization. The lactide monomers form a building chain, and the metal catalyst stops the lactide monomers from reacting back on the growing, nascent polymer chain [42]. The zinc metal catalyst thus helps to increase the rate of PLA polymerization of lactide intermediates with efficient chemical reaction directionality per Le Chatelier’s Principle.

After the polymerization process is complete, the PLA is dissolved in an organic solvent such as dichloromethane [Macron Fine Chemicals, cat. no. 1593, Center Valley, PA, USA] or chloroform [VWR Chemicals, cat. no. BDH1109-4LG, Radnor, PA, USA] under high temperatures (~100–120 °C). The dissolved PLA is then subjected to a stream of methanol [VWR, cat. no. BDH20864.400, Radnor, PA, USA], which causes the polymer to rapidly precipitate out of solution (Figure 4D). During this process, traces of the metal catalyst are also filtered out. The precipitated polymer is collected and then subjected to a process of evaporation to remove the methanol over the course of 4 h (Figure 4E). This is typically done using a regulated convection oven [Precision Scientific Group, model 18EM, cat. no. 31580, Winchester, VA, USA], which allows for the evaporation of the methanol while leaving behind a pure-grade polymer of PLA with a high molecular weight (Figure 4E). The use of a metal catalyst and organic solvents, along with high temperatures and vacuum conditions, allows for the efficient and controlled polymerization of PLA in vitro (Figure 4A–E). To characterize our PLA production quantity and quality, we employed ^1^H (protium) nuclear magnetic resonance (NMR) spectroscopy using a Bruker 400 megahertz (MHz, Billerica, MA, USA) in PLA samples dissolved in deuterated chloroform (CDCl_3_) solvent. Stereochemistry refers to the spatial arrangement of atoms in a molecule, and in the case of PLA, it refers to the arrangement of the lactic acid monomers in the polymer chain with respect to known functional groups. By monitoring the chemical shifts of the protons and carbons inside the molecule, NMR provides spatial details to evaluate the stereoregularity and stereosequence distribution of the harvested polymer.

### 2.8. Impregnation of PLA Disks with TMP

**Figure 5:**
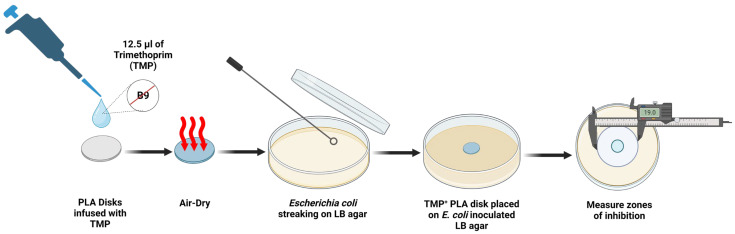
Antimicrobial drug elution through PLA disks. Created with Biorender.com (accessed on 29 January 2024).

After pouring the molten PLA onto a sterile petri plate and letting it cool for 10 min, we fashioned smaller disks from the slab of semi-solid PLA polymer with a height of less than 1 mm thickness. All disks were fashioned under sterile BSL-2 hood conditions [NuAire Labgard Class II Type A2 Biological Safety Cabinet, cat. no. NU-540, Plymouth, MN, USA]. We used a sterile 1 mL micropipette tip end as a punch-mold to quickly form PLA disks with a diameter (*D*) of 6 mm Batches of circular PLA disks (1 mm H × 6 mm *D*) were next collected in glass Petri dishes and each batch was desiccated in a convection oven [Precision Scientific Group, model 18EM, cat. no. 31580, Winchester, VA, USA] for 4 h at 40 °C. Limiting moisture content is important to stabilize PLA disk integrity and prevent PLA disk degradation over time. Additionally, drug concentrations impregnated into dry PLA disks are not diluted in downstream pharmacokinetic assays (Figure 5).

The melt casting method for forming PLA discs involves heating the PLA above its glass transition and melting temperatures to create a viscous liquid, which can then be shaped into films or disks [43,44,45]. The typical temperatures for PLA melt casting are around 160–180 °C [45]. As the PLA cools, it can either crystallize or remain amorphous depending on the cooling rate and the specific grade of PLA used [43,44,45]. Rapid cooling tends to result in more amorphous structures, while slower cooling allows for crystallization. The crystallinity or amorphicity of the polymer significantly affects the microstructure, which in turn influences properties like mechanical strength, degradation rate, etc., [45].

The crystalline structure of PLA disks formed using the process described in the paper depends on several factors, including the cooling rate and the temperature at which the disks are desiccated. The PLA is initially in a molten state and then allowed to cool for 10 min on a Petri plate. This cooling rate is relatively rapid, which tends to favor the formation of an amorphous structure rather than a highly crystalline one. After the disks are formed, they are desiccated at 40 °C for 4 h. This temperature is below the glass transition temperature (Tg) of PLA, which is typically around 60–65 °C. Desiccating below Tg usually does not significantly increase crystallinity [43,44,45].

Based on these conditions, the PLA disks are likely to have a predominantly amorphous structure with limited crystalline regions [45]. The amorphous nature could be beneficial for certain applications, such as drug impregnation and release, due to the increased free volume and potentially more accessible surface areas in amorphous polymers compared to their crystalline counterparts. However, exact crystallinity would need to be confirmed through analytical techniques like differential scanning calorimetry (DSC) or X-ray diffraction (XRD), which we did not perform.

Yet, the process of desiccation at 40 °C for 4 h could potentially lead to the formation of surface cracks in the PLA disks, especially if the moisture content is significant [46]. This is due to the differential shrinkage that occurs as the material dries and water evaporates. If the drying is uneven or too rapid, it can create stress within the material, leading to crack formation [46]. The extent and size of these cracks would depend on factors such as the initial moisture content, the uniformity of the drying process, and the physical properties of the PLA used. Surface cracks, if present in large quantities, can affect the mechanical integrity and surface area of the disks, potentially influencing drug loading and release properties.

Surface properties can indeed be more crucial for certain applications than the bulk material properties. In the context of packing TMP (trimethoprim) on PLA (poly-lactic acid) substrates, surface characteristics like surface tension and the presence of micro-cracks or surface roughness can significantly influence the deposition process. These surface features can affect how molecules interact with the substrate, potentially overriding the influence of the crystalline or amorphous nature of the bulk material. Therefore, focusing on the surface properties of PLA, rather than its bulk crystallinity, could be more relevant for understanding and optimizing TMP deposition.

### 2.9. Elution of Antimicrobials

**Figure 6:**
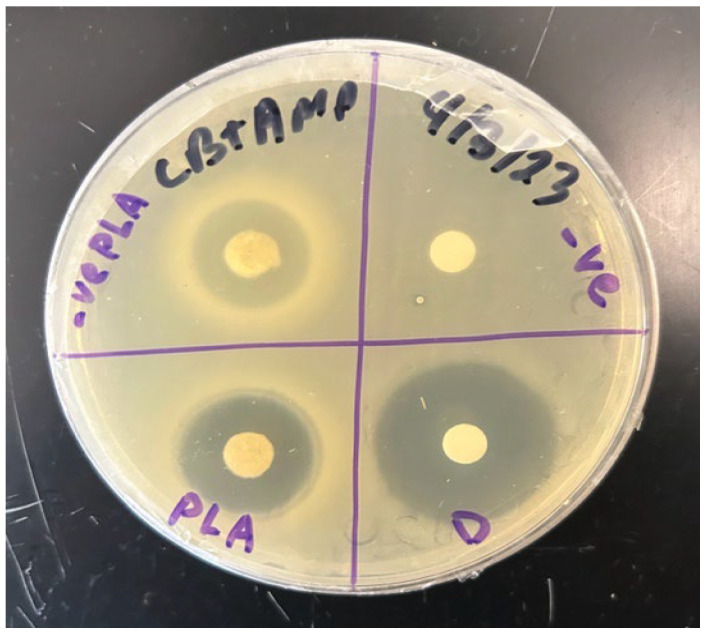
Elution of TMP from control and PLA disks.

Antimicrobials are a broad class of drugs that include naturally made antibiotics versus artificially synthesized chemotherapeuticals. Most antimicrobials, including the sulfa chemotherapeutical drug known as trimethoprim (TMP), are heat-labile and cannot be autoclaved. Each antimicrobial also exhibits a different minimal inhibitory concentration (MIC) for any given bacterial species, and this MIC can be determined using standard Kirby–Bauer disk diffusion or use-dilution assays (Figure 5). A drug’s MIC value thus is an optimal drug concentration that leverages antimicrobial killing power with minimal toxic side effects in the host. Trimethoprim (TMP) [VWR, cat. no. AAJ63053-03, Radnor, PA, USA] was impregnated into our PLA disks using the following procedure. Stock TMP solutions were prepared in aqueous solvent at maximum solubility concentrations of 0.4 mg TMP per mL water, and then filter-sterilized using 50 mL Luer-Lok tip syringes [BD Biosciences, cat. no. 309653, Franklin Lakes, NJ, USA] using 0.2 µm Whatman GD/X pores [Cytiva, cat. no. 6896-2502, Marlborough, MA, USA]. Literature reviews indicate that typical MIC values for TMP occur at 0.05 µg per mL for *E. coli* [47]. Standardized TMP disks are routinely accessed by the Clinical & Laboratory Standards Institute (CLSI) located in Wayne, PA, USA and the most recent, standardized TMP amount is 5 µg of TMP per CLSI-compliant disk [48]. Desiccated PLA disks (1 mm H × 6 mm *D*) were carefully placed on sterile plastic petri dish edges and 12.5 µL of stock 0.4 mg/mL TMP were carefully loaded to each individual PLA disk using a micropipette in a sterile hood [NuAire Labgard Class II Type A2 Biological Safety Cabinet, cat. no. NU-540, Plymouth, MN, USA] to yield 5 µg TMP-infused PLA disks. Each TMP-PLA disk was then gently dried at room temperature (24 °C) under sterile conditions in the bacteriological hood for 2 h total (1 h per disk side). Control disks were similarly fashioned using sterile 6 mm cloth disks [BD Biosciences, cat. no. 231039, Franklin Lakes, NJ, USA] that were TMP-infused in parallel to the PLA disk treatment. Dried PLA disks can then be tested for eluting impregnated TMP drug in a modified Kirby–Bauer assay on LB + ampicillin [working 75 µg/mL, VWR, cat. no. 100219-890, Radnor, PA, USA] agar plates that were lawn-inoculated with ampicillin-resistant *E. coli* (Migula) Castellani and Chalmers strain [ATCC, cat. no. 700891, Manassas, VA, USA]. The TMP molecules disperse from the polymeric PLA matrix into the surrounding LB nutrient agar as explained by diffusion coefficients and Fick’s law of mass transfer [49]. During the course of the Kirby–Bauer assay, ZOIs were measured across a 10 h timeframe, at 1 h time intervals (Figure 6).

## 3. Results

In this exploratory and feasibility study, the fermentation of raw materials was conducted using 500 g of beer spent grain (BSG) with 2000 mL of water. The fermentation process resulted in the production of 500 mL of crude lactic acid (LA), which was reduced to 300 mL after filtration, as described in Section 2.5. The concentration of the filtered LA was determined to be 40% in mass, as measured by a refractometer [50], which provided a refractive index for the solution of 1.38.

The relationship between the refractive index of lactic acid in an aqueous solution and its mass fraction has been shown to vary linearly, as referenced from prior experimentation [50]. This linear relationship was instrumental in determining the concentration of lactic acid in the solution. The yield of PLA from the synthesis process was 50%. While the molecular mass of the material was not directly measured, the synthesis process using zinc as a catalyst and dichloromethane as a solvent is known to provide a low molecular mass after polymerization. As a reference [51], the use of a zinc catalyst in CH_2_Cl_2_ at a temperature of 160 °C provided a molecular weight of 35,000 g/mol.

These results provide valuable insights into the effectiveness of using BSG as a raw material for PLA production. The yield of lactic acid, in terms of weight from the BSG, can be inferred from the concentration and volume of the final PLA product, demonstrating the potential of this method for sustainable material production.

**Figure 7:**
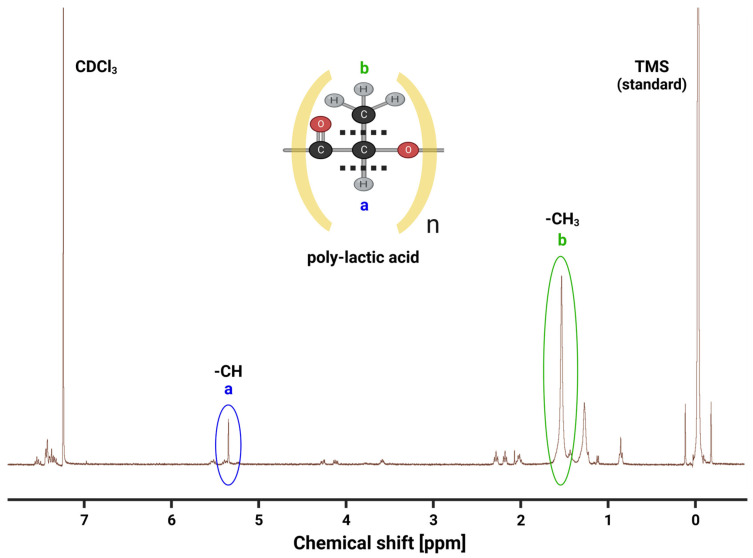
NMR analysis of PLA purity in chloroform. (**a**) The minor peak corresponds to the -CH group’s chemical shift. (**b**) The larger peak characterizes the methyl (-CH_3_) group that branches off the core, repeating unit of the poly-lactic acid.

Nuclear magnetic resonance (NMR) spectroscopy was used to analyze PLA polymer production efficacy qualitatively and quantitatively (Figure 7). NMR effectively determines a substance’s electromagnetic signal near resonance and is useful for identifying molecular signatures comprising patterns of functional groups. When the PLA sample in a deuterated chloroform (CDCl_3_) carrier is loaded into the ^1^H NMR spectrometer (400 MHz), a spectrum of peaks that correspond to specific magnetic fields is obtained. These peaks can then be analyzed to determine the types and locations of different atoms within the PLA molecule.

One important stereosequence feature of the NMR spectrum of the PLA sample is the presence of peaks at 1.6 ppm and 5.3 ppm [52], which correspond to the methyl group and methine protons of the PLA monomer, respectively (Figure 7). These peaks provide identifying characteristics suggesting that our lactic acid harvest and PLA polymerization methodology is indeed replete with PLA molecular signatures. Another peak that appears in the NMR spectrum of the PLA sample is located at 7.26 ppm [53], which corresponds to the chloroform solvent used in the sample preparation for NMR operations (Figure 7). The relative peak magnitudes at 1.6 ppm and 5.3 ppm together qualitatively suggest that our polymerization reactions are yielding high-grade, refined PLA polymers.

The NMR spectrum of the PLA sample also shows a few additional peaks between 3.5 ppm and 4.5 ppm, which likely suggest the presence of impurities or degradation products in the sample that remained after filtration attempts. However, the relative abundances of PLA-specific peak signatures compared to impurity peaks indicates that our PLA production method does generate a relatively high quality of polymer. This information is important for ensuring the quality and consistency of the PLA polymer, which is critical for its use in various industrial and biomedical applications.

**Figure 8:**
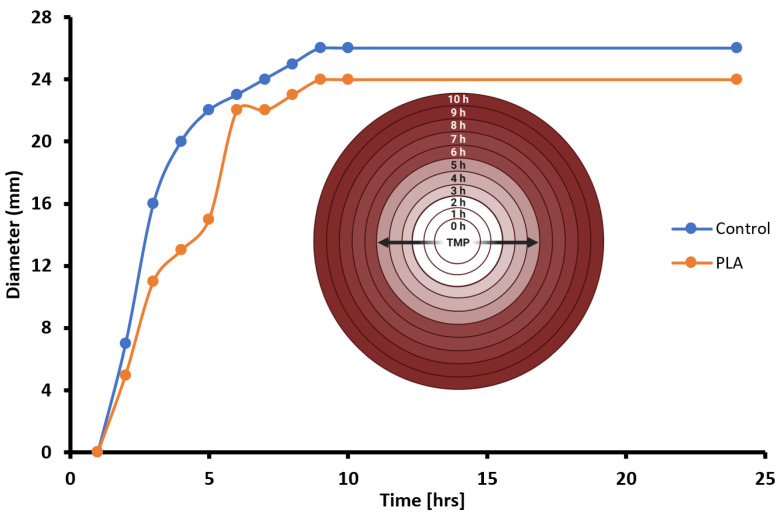
Antimicrobial Susceptibility to TMP Delivery by PLA.

To investigate the drug-elution properties of poly-lactic acid (PLA) disks, a modified Kirby–Bauer assay was conducted in which the TMP-infused PLA disks were placed on LB + ampicillin agar plates that were lawn-inoculated with pure cultures of *E. coli* that contained the *bla* operon that encodes beta-lactamase for ampicillin resistance. For comparison, a control group of sterile paper disks impregnated with identical amounts of TMP were seeded near the TMP^+^ PLA experimental disks. After overnight incubation (8–12 h) at 37 °C (i.e., the optimal growth temperature of *E. coli*), LA + amp plates were examined for the presence of zones of inhibition (ZOIs) indicative of the effective TMP-mediated killing of *E. coli* (Figure 5). All ZOI diameters (in mm) were measured at several timepoints (Figure 6). To visualize the changing kinetics of TMP delivery into the nutrient media, we graphed ZOIs from both PLA and control disks over a 10 h period with a temporal resolution of 1 h intervals (Figure 8). Generally, both PLA and control disks qualitatively approximate each other in terms of TMP drug delivery into nutrient agar media containing ampicillin-resistant, TMP-sensitive *E. coli*. Further, PLA disk delivery of TMP arrives at similar ZOI saturation responses at ~9 h and such responses are likely driven by both diffusion rate kinetics overlaid on top of microbial susceptibility parameters such as folate metabolism interference by TMP in *E. coli*. Prior to ZOI saturation responses, a qualitative assessment suggests that both PLA and control disks exhibit non-linear drug effusion rates inflection points with the greatest tangential ZOI slopes at ~2 h into the response (Figure 8). However, after 7 more hours, the PLA disks began to show a similar killing radius to that of the control disks. This suggests that while the PLA disks had a delayed response, they ultimately were able to inhibit bacterial growth to a similar extent as the control disks, as both delivery systems reached antimicrobial saturation limits.

The minimum inhibitory concentration (MIC) of TMP has been empirically determined to be 0.05 µg/mL in the literature for *E*. *coli* [47]. Although subtle, a developing ZOI over time is driven by numerous factors, including TMP diffusion kinetics during elution waves from PLA disks layered over microbial concerns such as sufficient TMP drug blockade of bacterial dihydrofolate reductase to prevent microbial folate anabolism and thus inhibit the thymidine synthesis needed for DNA replication preceding binary fission. When the TMP drug diffuses from the disk, it inhibits bacterial growth and creates a developing zone of inhibition. The final size of the zone of inhibition for drug-sensitive microbes varies with the concentration of the antimicrobial initially infused into the disk. It is assumed that the minimum amount of TMP required to create a zone of inhibition is also the minimum amount of antimicrobial that needs to be released from the polymer matrix of the PLA disk to minimally suppress folate metabolism. If the concentration of TMP in the disk or any diffusion vectors emanating from the disk falls below the MIC, there will be no zone of inhibition. As a finite amount of TMP drug is loaded into each PLA disk, during the course of the Kirby–Bauer assay, the concentration of TMP in the disk decreases as the antimicrobial diffuses away, which terminates the expansion wave of the ZOI. Under these conditions, any *E*. *coli* cells at the TMP diffusion wavefront will experience sub-optimal killing as concentrations of TMP fall below known MIC values.

**Figure 9:**
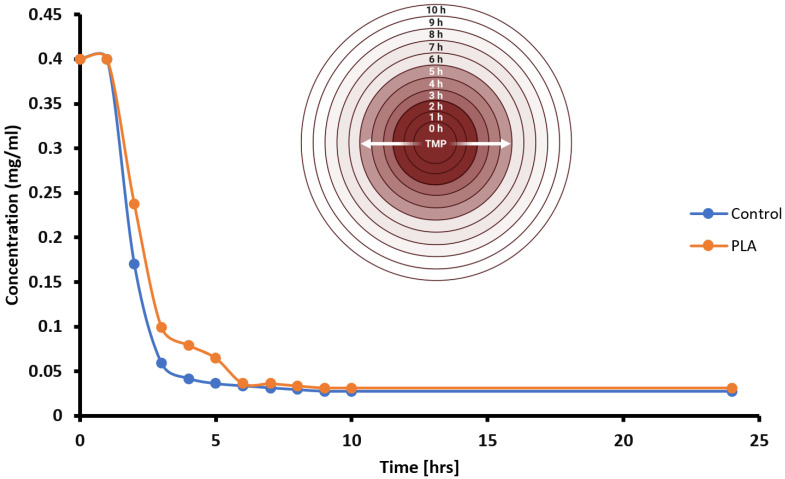
Estimated TMP Diffusion Kinetics Using Data-Transformed ZOIs.

Knowledge of the loaded amount of TMP as well as the literature-derived TMP MIC value enables us to transform the empirically observed ZOI dataset as an indirect measurement of TMP diffusion kinetics. A graph was plotted to illustrate the estimated concentration of TMP diffusion through biological samples over time (Figure 9). Initially, both experimental and control disks were loaded with equivalent mass amounts of the TMP drug (5 µg), and, over a 10 h period, data transformation plots indicated four general wavefronts (W_1_–W_4_) of TMP diffusion, with rapid diffusion seen in the first two hours (first wavefront, W_1_), followed by gradual decrements in TMP diffusion rates in subsequent wavefronts (W_2_–W_4_). Qualitatively, the irregular curve for the PLA group suggested the presence of trace impurities in our PLA harvest and polymerization methodologies (Figure 9).

## 4. Discussion

This study presents a promising approach to producing a biodegradable polymer (i.e., PLA) from Brewer’s spent grain (BSG) that has important implications for the medical and pharmaceutical industry. The PLA can be used as a drug delivery system or as a scaffold for tissue engineering, and the Kirby–Bauer test results here show that our PLA formulation succeeded at releasing antimicrobial drugs. As the most common biomaterial for orthopedic strategies is PMMA, available drugs for elution must be heat-resistant (i.e., tobramycin or vancomycin) since PMMA exothermic reactions for polymerization range from 100–140 °C, depending on curing times [54]. We purposefully chose a heat-labile antimicrobial (i.e., TMP) to demonstrate PLA’s intrinsic ability to elute a drug that is normally inaccessible to PMMA polymerization technologies. Thus, our PLA approach expands the pharmacopeia landscape by including all drugs across all temperature sensitivities. As methicillin-resistant *Staphylococcus aureus* (MRSA) strains exhibit multi-drug resistance (MDR) phenotypes, MRSA, as a common nosocomial agent of orthopedic surgical infections, requires an enhanced repertoire of available antimicrobials, including antibiotics, semi-synthetics, and chemotherapeuticals to resolve infections. Overall, this approach can reduce post-surgical complications involving MRSA, and contribute to the development of new PLA-centric biomaterials with important medical applications.

Overall, the study highlights the potential of upcycling discarded materials in the form of BSG to produce valuable biomaterials of medical importance. We envision PLA-based biomaterials impregnated with a larger palette of available drugs to enhance host healing and self-renewal. PLA in turn can be fashioned into various biodegradable form factors for use in medical sutures, hydrogel-based bandages, and scaffolds for grafting operations in tissue engineering. In contrast, PMMA-based biomaterials are not biodegradable and require subsequent removal following host renewal. Future research in this field could thus lead to the development of new PLA-inspired biomaterials with improved properties and applications in a variety of medical fields.

## 5. Conclusions

Taken together, these findings are very encouraging for future exploration of PLA-based biomaterials in drug delivery applications. Further research is needed to fully optimize the entire PLA production pipeline to maximize biomaterial output, and polymer quality devoid of impurities. PLA-based drug delivery systems offer many advantages over conventional strategies, especially given the prevalence of multi-drug-resistant and extreme-drug-resistant bacterial agents of disease that permeate the modern hospital environment.

## Data Availability

The data presented in this study are available on request from the corresponding author. The data are not publicly available due to terms and policy.
